# Genetic evidence that high BMI in childhood has a protective effect on intermediate diabetes traits, including measures of insulin sensitivity and secretion, after accounting for BMI in adulthood

**DOI:** 10.1007/s00125-023-05923-6

**Published:** 2023-06-06

**Authors:** Gareth Hawkes, Robin N. Beaumont, Jessica Tyrrell, Grace M. Power, Andrew Wood, Markku Laakso, Lilian Fernandes Silva, Michael Boehnke, Xianyong Yin, Tom G. Richardson, George Davey Smith, Timothy M. Frayling

**Affiliations:** 1 Genetics of Complex Traits, College of Medicine and Health, University of Exeter, Exeter, Devon UK; 2 MRC Integrative Epidemiology Unit, Population Health Sciences, Bristol Medical School, University of Bristol, Bristol, UK; 3 School of Medicine, Institute of Clinical Medicine, University of Eastern Finland, Kuopio, Finland; 4 Department of Biostatistics and Center for Statistical Genetics, University of Michigan School of Public Health, Ann Arbor, MI USA

**Keywords:** Adulthood, BMI, Childhood, Diabetes, Genetics, Mendelian randomisation

## Abstract

**Aims/hypothesis:**

Determining how high BMI at different time points influences the risk of developing type 2 diabetes and affects insulin secretion and insulin sensitivity is critical.

**Methods:**

By estimating childhood BMI in 441,761 individuals in the UK Biobank, we identified which genetic variants had larger effects on adulthood BMI than on childhood BMI, and vice versa. All genome-wide significant genetic variants were then used to separate the independent genetic effects of high childhood BMI from those of high adulthood BMI on the risk of type 2 diabetes and insulin-related phenotypes using Mendelian randomisation. We performed two-sample MR using external studies of type 2 diabetes, and oral and intravenous measures of insulin secretion and sensitivity.

**Results:**

We found that a childhood BMI that was one standard deviation (1.97 kg/m^2^) higher than the mean, corrected for the independent genetic liability to adulthood BMI, was associated with a protective effect for seven measures of insulin sensitivity and secretion, including increased insulin sensitivity index (β=0*.*15; 95% CI 0.067, 0.225; *p*=2.79×10^−4^) and reduced fasting glucose levels (β=−0.053; 95% CI −0.089, −0.017; *p*=4.31×10^−3^). However, there was little to no evidence of a direct protective effect on type 2 diabetes (OR 0.94; 95% CI 0.85, 1.04; *p*=0.228) independently of genetic liability to adulthood BMI.

**Conclusions/interpretation:**

Our results provide evidence of the protective effect of higher childhood BMI on insulin secretion and sensitivity, which are crucial intermediate diabetes traits. However, we stress that our results should not currently lead to any change in public health or clinical practice, given the uncertainty regarding the biological pathway of these effects and the limitations of this type of study.

**Graphical Abstract:**

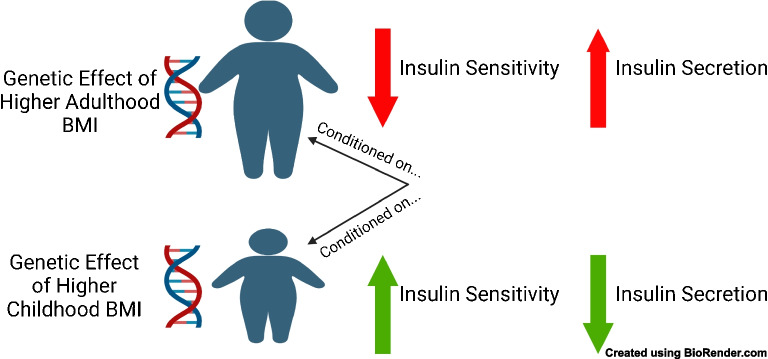

**Supplementary Information:**

The online version of this article (10.1007/s00125-023-05923-6) contains peer-reviewed but unedited supplementary material.


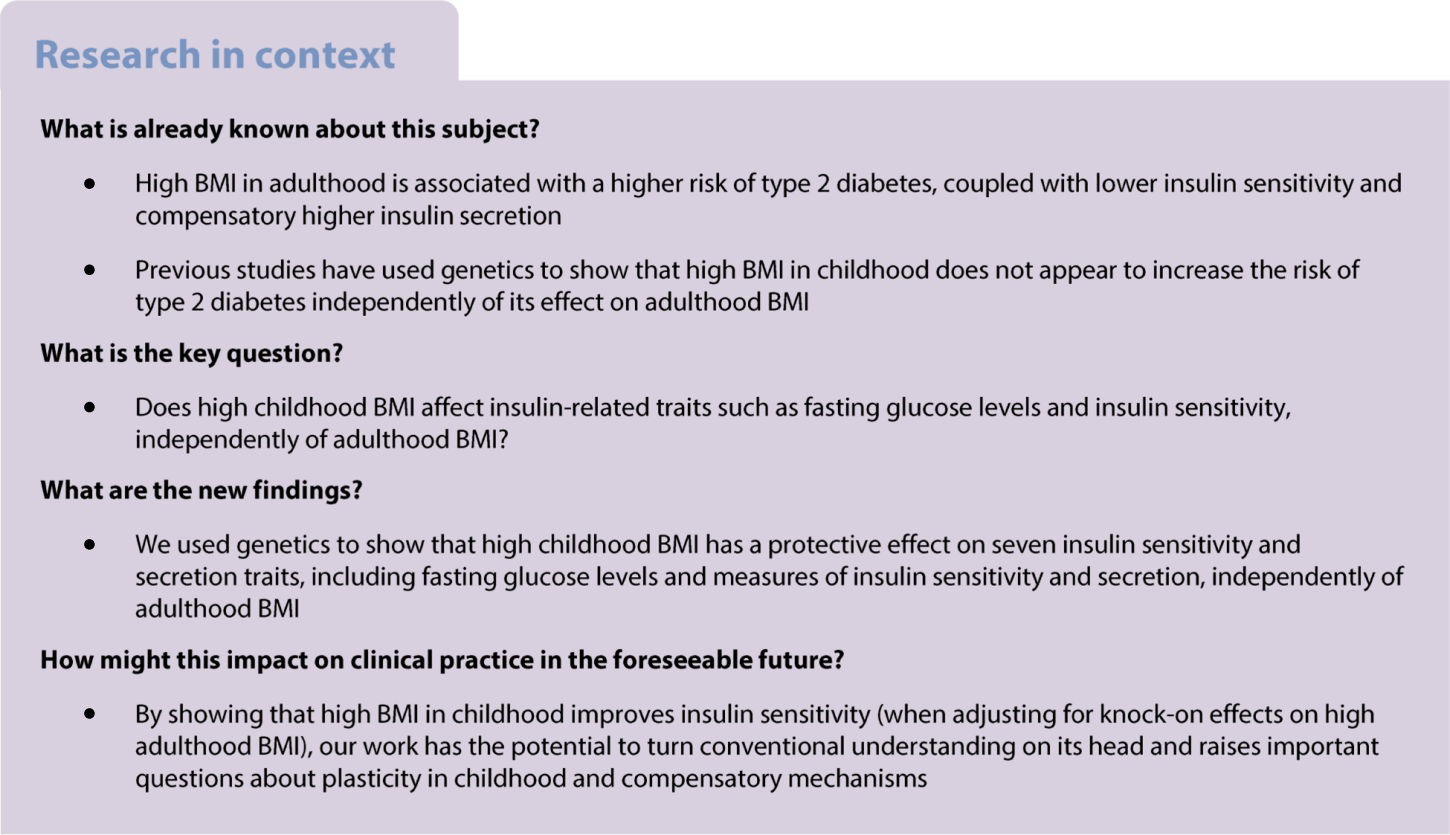


## Introduction

The increasing prevalence of obesity in childhood is assumed to lead to an increased prevalence of type 2 diabetes in adult life [1]. Previous observational studies have shown that changing from a relatively thin child to an overweight or obese adult provides an additional risk for type 2 diabetes, compared with current adulthood BMI [2]. However, observational studies are subject to confounding, which is less likely to affect genetic studies [3]. For example, an unmeasured factor, such as smoking status, may confound the association between observed BMI and diabetes status, but cannot affect the genetic variants that an individual carries.

A previous study that used genetics to understand the causality of higher BMI at different time points on type 2 diabetes found that the relationship between childhood BMI and type 2 diabetes was mediated through adulthood BMI [4]. The study used genetic variants with stronger effects on adulthood BMI than childhood BMI, and vice versa, to separately test the effects of high BMI in childhood and the effects of high BMI in adulthood. However, the study was limited to analysis of type 2 diabetes as a binary disease trait, and did not investigate potential intermediate mechanisms such as those involving insulin secretion and sensitivity; in addition, the genetic analysis was limited to lower-powered categorical BMI phenotypes. To understand more about the relationship between higher childhood BMI and type 2 diabetes, we generated a continuous measure of childhood BMI in the UK Biobank, validated using the 1958 National Childhood Development Study (1958NCDS) [5], which can be directly compared with continuous adulthood BMI, resulting in a more powerful genetic approach. We then tested a wide range of intermediate diabetes risk factors.

Using a combination of both previously identified instruments and novel genetic instruments for childhood and adulthood BMI that resulted from our continuous phenotypes, we assessed the causal relationships between BMI at different life stages and diabetes and related outcomes: type 2 diabetes, fasting insulin (FI) levels, fasting glucose (FG) levels, and several measures of insulin secretion and sensitivity based on oral and intravenous tests, using multivariable Mendelian randomisation [6].

## Methods

### Study population

We analysed 441,761 individuals of inferred European descent within the UK Biobank based on genetic principal component analysis, as previously described [7], using imputed genome-wide genetic variants, and both a baseline (adulthood) BMI measure (UKB Field 21001) and a self-recall variable related to body size at age 10 years (UKB Field 1687).

### 1958 National Child Development Study

The 1958NCDS is a longitudinal assessment of 17,415 individuals who were born within a single week in March 1958 [5]. Beginning at the week of birth, mothers and their children were repeatedly assessed at irregular intervals, using a comprehensive set of measurements and assessments regarding many aspects of their lives. We analysed a subset of 5847 individuals who had undergone genome-wide array-based genotyping and imputation, and who were inferred to be of European descent (again using genetic principal component analysis). Of these 5847 individuals, 4838 had measures of BMI at age 7 years, 4704 at age 11 years, 4298 at age 16 years, 5013 at age 23 years, and 5774 at age 44 years.

### Measures of BMI

Individuals in the UK Biobank were asked whether they felt that they were ‘thinner’, ‘the same size as’ or ‘plumper’ than their peers when they were age 10 years (UKB Field 1687). From this categorical variable, we generated a continuous simulation of childhood BMI in the UK Biobank based on summary statistics for BMI at age 11 years from the 1958 National Child Development Study (see electronic supplementary material [ESM] Methods for full details, and ESM Figs 1–3, respectively). Briefly, the self-recall value for each participant’s body size at age 10 years was used as an anchor to which we assigned a BMI at age 10 years, after sub-sampling from a distribution that was an approximation of BMI at age 11 years in the 1958NCDS. Next, using the UK Biobank data, a genome-wide association study (GWAS) was performed for both adulthood and childhood BMI, from which we generated two genetic instruments. Adulthood BMI was taken directly from UKB Field 21001.

### Genetic variants associated with BMI

We used the software REGENIE [8] to assess the association between each of 65,433,624 imputed genetic variants and BMI at each of the two timepoints independently for 441,762 individuals. We then excluded genetic variants that were not SNPs, and those that did not have INFO >0*.*8 and minor allele frequency (MAF) of 0.01<MAF<0.99. REGENIE performs association tests using a linear mixed-model approach, which takes account of the degree of genetic relationship between each pair of individuals. The BMI at both timepoints was rank inverse-normalised and residualised at run time: the covariates adjusted for were sex, age at baseline, genotyping chip and UK Biobank assessment centre. Effect sizes are given in standard deviation units.

Based on the results of these GWAS, we used the clumping procedure in PLINK version 1.9 [9] to select independent and genome-level associated genetic variants. We used the following criteria to define an independent genetic association: *r*^2^≤0*.*001 (correlation between independent signals), distance ≥250 kb (distance between independent signals), *p*≤5×10^−8^ and 0*.*01≤MAF≤0*.*99, using an unrelated quality-controlled HapMap3 reference panel [10].

### Validation of genetic scores

The independent genetic variants for childhood and adulthood BMI derived here were individually assessed against phenotypes available in the 1958NCDS. A genetic risk score for both adulthood BMI and BMI at age 10 years was calculated within the 1958NCDS cohort using the variants identified from the relevant GWAS, and assessed against derived BMI for each individual at the age of 44 years and 11 years. To make a direct comparison between the predictive ability of the two genetic risk scores against the two phenotypes, we calculated the receiver–operator curves for the accuracy in predicting one of the two phenotypes being greater than one standard deviation from the standardised mean of the phenotype. We additionally calculated the percentage of the variance explained by the continuous genetic score against the phenotype of interest.

At a genome-wide level, we calculated the genetic correlation of both adulthood and childhood BMI with both the most recent Early Growth Genetics (EGG) Consortium meta-analysis of childhood BMI [11] and adulthood BMI [12]. We used the R software package *LDSC* to calculate genetic correlation [13]. Finally, we calculated the total variance explained by the instruments for adulthood and childhood BMI separately using the following formula: 2 × β^2^ × MAF × (1 − MAF).

### Mendelian randomisation

We used Mendelian randomisation (MR) to assess whether there is a causal link [14] between our BMI exposures and type 2 diabetes/insulin-related outcomes. In an MR study, the effect sizes of independent genetic variants that are strongly associated with each exposure are regressed against the effect sizes of the same variants with the disease/outcome from a GWAS of a secondary non-overlapping cohort (two-sample MR). Comparison at a genetic level bypasses some observational confounders, as genetic variant genotypes are determined at zygote formation [14]. As such, an association found using MR provides stronger evidence of causality than that derived from observational data, albeit with the possibility of residual confounding if there is, for example, residual population structure.

We calculated the MR causal effect estimates using an inverse-variance weighted model, where each variant–exposure vs variant–outcome relationship is weighted by the inverse of the variance of the variant–outcome relationship. A sensitivity analysis was also performed in a lower-power MR–Egger framework, which is more robust to pleiotropy (an association between the variant and outcome that does not pass through the exposure, which is a violation of the MR assumptions). Additionally, we performed a sensitivity analysis with Steiger filtering applied [15]. This approach excludes genetic variants that have larger effects on the outcomes (such as insulin secretion and sensitivity measures) than the exposures (childhood or adulthood BMI). We calculated effects using the formula used to compare instrument strength in the previous section. We also calculated an *F*-statistic for each of our analyses as a measure of the quality of the variants as a genetic proxy for the observed exposure (typically *F*>10 is classified as sufficient).

Where the variant–outcome relationship was not available in the outcome GWAS, a variant proxy was chosen based on a high degree of correlation (*r*^2^>0*.*8) between the index variant and its proxy and a maximum distance between the index and proxy variant of 250 kbp. Effect sizes between the variant and outcome and variant–exposure were then either orientated to the matching alleles or matched based upon the reported allele frequencies.

**Fig. 1:**
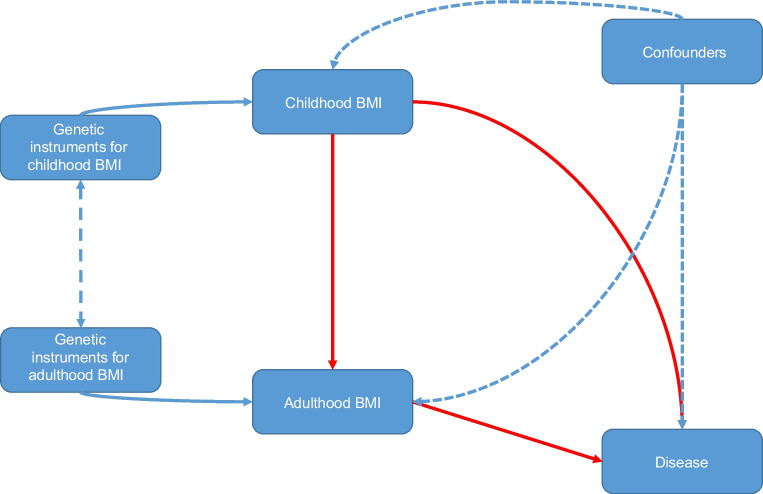
Directed acyclic graph illustrating assumed causal relationship between childhood and adulthood BMI. Solid red and blue arrows indicate causality, unidirectional blue dashed arrows represent non-causal effects, and the double-ended blue dashed arrows indicate a covariance structure

Multivariable Mendelian randomisation (MVMR) is performed by conditioning the exposure–outcome relationship for each genetic variant upon that of another exposure’s genetic effect size (for example, conditioning the exposure–outcome relationship of childhood BMI upon that of adulthood BMI [6]). As such, the adjusted primary exposure–outcome relationship is independent of the genetic effects associated with the secondary exposure; refer to Fig. 1 for a pictorial representation of the MVMR model.

### Measures of type 2 diabetes and insulin-related traits

Genetic variant effect sizes for type 2 diabetes were obtained from a previous study [16], which was a meta-analysis of 71,124 cases and 824,006 controls of European ancestry, and from FinnGen (Freeze Six) [17] for ‘Type 2 diabetes, strict (exclude DM1)’, which included 37,002 cases and 215,160 controls. The MR results for each type 2 diabetes-outcome GWAS were then meta-analysed.

Effect sizes relating to FG and FI levels were drawn from a previous study [18], in which FG levels were measured in 151,188 individuals and FI levels were measured in 105,056 individuals.

We also analysed seven measures of insulin sensitivity and response during and after an oral glucose tolerance test in a meta-analysis of results from a study of 26,037 participants without diabetes [19] and the Metabolic Syndrome in Men (METSIM) study [20], which included male participants from Kuopio, a town in Finland. Specifically, we looked at the association of our BMI measures with the area under the insulin curve (AUC), the ratio of the AUCs for insulin and glucose (AUC ratio), an insulin sensitivity index, insulin after 30 min, insulin after 30 min adjusted for BMI, incremental insulin after 30 min and corrected insulin response, as defined previously [19].

Meta-analyses were performed, where applicable, using the R software package *metafor*, based on the assumption of a fixed effect between the exposure and outcome across studies.

## Results

In our analysis of 441,762 adult individuals (aged 40–75 years), we identified 306 (ESM Table 1) and 1127 (ESM Table 2) independent genetic variants (*p*<5×10^−8^, 250 kb distance) associated with continuous measures of childhood and adulthood BMI, respectively, in comparison with a previous study [4], in which 295 and 557 independent signals, respectively, were reported for categorical measures of the same outcomes. The exact parameters derived to describe the continuous measure of childhood BMI are given in the ESM Results. The variance explained by the genetic variants was 11.3% and 4.03% for adulthood and childhood BMI, respectively, compared with 2.78% and 1.96% in the previous study [4], demonstrating that our genetic instruments have been strengthened by using continuous variables.

Using these variants, we generated polygenic scores for childhood and adulthood BMI, and validated them in the 1958NCDS dataset and independent data from the EGG Consortium [11] and the Genetic Investigation of ANthropometric Traits (GIANT) Consortium [12]. The adulthood BMI genetic risk score was a better predictor of standardised adulthood BMI at age 44 years in the 1958NCDS dataset (OR for being greater than one standard deviation from the mean 1.55; 95% CI 1.43, 1.67;*p*=1.29×10^−16^; variance 4.80%) than of standardised childhood BMI at age 11 years being more than one standard deviation from the mean in the same dataset (OR 1.32; 95% CI 1.20, 1.44; *p*=1.85×10^−9^; variance 0.995%), and explained more of the variance in the respective continuous traits (ESM Fig. 4). The childhood BMI genetic risk score was a better predictor of childhood BMI (OR 1.32; 95% CI 1.21, 1.44; *p*=1.32×10^−16^; variance 1.62%) than of adulthood BMI (OR 1.16; 95% CI 1.08, 1.25; *p*=3.73×10^−5^, variance 0.635%).

The genetic correlation between adulthood BMI measured in this UKBiobank study and childhood BMI measured directly by the EGG Consortium (*n*=35,668, ages 2–10 years) was 0.644 (95% CI 0.582, 0.705; *p*=3.22×10^−95^), which was less than that between our measure of childhood BMI and that of the EGG Consortium, at 0.937 (95% CI 0.864, 1.01; *p*=1.29×10^−145^). The genetic correlation between adulthood BMI analysed by the GIANT Consortium [12] and adulthood BMI measured in this UK Biobank study was 0.943 (95% CI 0.925, 0.960; *p*=1.31×10^−210^), and the genetic correlation with childhood BMI measured here was 0.645 (95% CI 0.591, 0.680; *p*=1.28×10^−173^).

### Childhood BMI

Using MR, we showed that higher BMI in childhood was associated with protective effects on diabetes-related traits after adjustment for the independent effects of higher adulthood BMI. Specifically, MR showed that higher childhood BMI, corrected for genetic liability to adulthood BMI, has protective effects on insulin secretion and sensitivity traits, but that higher childhood BMI, corrected for genetic liability to adulthood BMI, was not protective for type 2 diabetes.

**Fig. 2:**
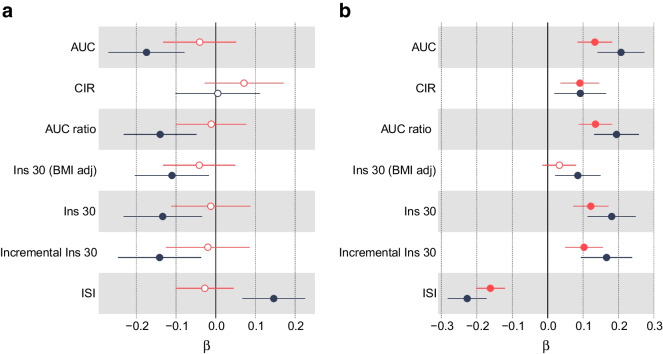
Univariable MR meta-analysis results (red lines and symbols) and MVMR meta-analysis results (blue lines and symbols) for (**a**) childhood BMI and (**b**) adulthood BMI vs oral glucose tolerance test traits: AUC; corrected insulin response (CIR); ratio of insulin and glucose AUCs (AUC ratio); insulin after 30 min adjusted for BMI [Ins 30 (BMI adj)]; insulin after 30 min (Ins 30); insulin change after 30 min (incremental Ins 30); and insulin sensitivity index (ISI). Filled symbols indicate *p*<0.05

**Fig. 3:**
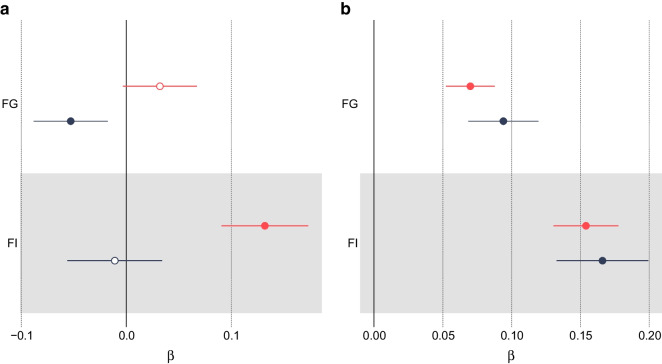
Univariable MR results (red lines and symbols) and MVMR results (blue lines and symbols) for the association between (**a**) childhood BMI and (**b**) adulthood BMI and FI and FG levels. Filled symbols indicate *p*<0.05

In a multivariable model that takes account of the independent genetic effects of adulthood BMI, we found that a childhood BMI that was one standard deviation (1.97 kg/m^2^) higher than the mean was associated with a protective effect on a range of insulin secretion and sensitivity traits: lower AUC, ratio of insulin and glucose curves (AUC ratio), insulin after 30 min adjusted for BMI, insulin change after 30 min, and higher insulin sensitivity index . Higher childhood BMI was also weakly associated with lower FG levels (β=−0.0528; 95% CI −0.0893, −0.0163; *p*=4.21×10^−3^). There was no evidence of an effect on FI levels (β=−0.0109; 95% CI −0.0564, 0.0346; *p*=0.638). These results, including a comparison with univariable models with no adjustment for adulthood BMI, are shown in Figs 2 and 3 and ESM Table 3.

**Fig. 4:**
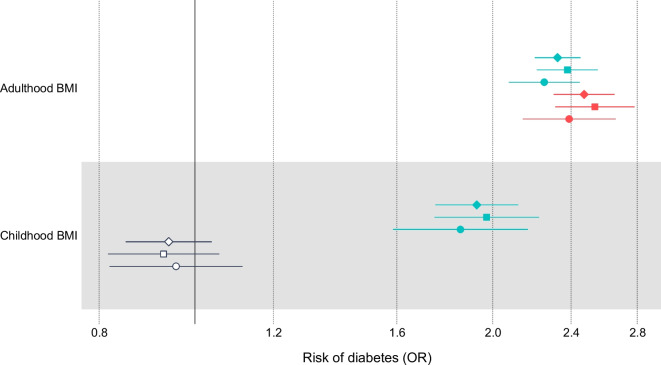
Univariable MR results (turquoise lines and symbols) and MVMR results (red/blue lines and symbols) for the association between childhood BMI and adulthood BMI and type 2 diabetes as measured in the present meta-analysis (diamonds), the study by Mahajan et al [16] (squares) and the FinnGen study [17] (circles). Filled symbols indicate *p*<0.05

In a multivariable model that corrects for the independent genetic liability to adulthood BMI, higher childhood BMI was not associated with the risk of type 2 diabetes (OR 0.941; 95% CI 0.851, 1.04; *p*=0.228), consistent with results derived from two meta-analysed studies [16, 17]. These results, including a comparison with the univariable model with no adjustment for adulthood BMI, are shown in Fig. 4 and ESM Table 3.

### Adulthood BMI

Using MVMR, we showed that higher BMI in adulthood leads to a higher risk of type 2 diabetes, independently of the genetic effects of childhood BMI. We observed consistent effects on insulin and glycaemic traits intermediate to type 2 diabetes, with MVMR showing that higher adulthood BMI leads to lower insulin sensitivity and higher insulin secretion. The link between higher genetically derived adulthood BMI and higher insulin secretion in people without type 2 diabetes is probably a response to lower insulin sensitivity. MR also showed that higher adulthood BMI was associated with an increased risk of type 2 diabetes.

In a multivariable model that corrects for the independent genetic liability to childhood BMI, an adulthood BMI that was one standard deviation (4.77 kg/m^2^) higher than the mean was associated with higher FG levels (β=0.0941; 95% CI 0.0683, 0.120; *p*=7.50×10^−13^) and FI levels (β=0*.*166; 95% CI 0*.*134, 0*.*199; *p*=3*.*19×10^−10^). An increase in adulthood BMI in a multivariable model also showed evidence of a damaging effect on the remaining six insulin traits (ESM Table 3). These results, including a comparison with the univariable model with no adjustment for childhood BMI, are shown in Figs 2 and 3 and ESM Table 3.

In a multivariable model that corrects for the independent genetic liability to childhood BMI, an adulthood BMI that was one standard deviation (4.77 kg/m^2^) higher than the mean was associated with an increased risk of type 2 diabetes (OR 2.47; 95% CI 2.31, 2.65; *p*=1.23×10^−142^). These results, including a comparison with the univariable model with no adjustment for childhood BMI, are shown in Fig. 4 and ESM Table 3.

### Sensitivity analyses

MR–Egger intercept analyses for each of the 57 MVMR models identified three statistical associations with *p*<0.01 for the following exposure–outcome relationships: FI from the MAGIC Consortium [18] (β=1.40×10^−3^, *p*=9.44×10^−4^), and type 2 diabetes from both the study by Mahajan et al [16] and the FinnGen study [17] (β=(4.00×10^−3^ and 4.00×10^−3^; and *p*=5.00×10^−3^ and 1.00×10^−3^, respectively; see ESM Table 4).

A Steiger-filtered analysis resulted in consistently wider confidence intervals compared with those obtained without filtering (ESM Table 5), but the results were broadly consistent. For example, we continued to observe evidence of a protective effect of higher childhood BMI, after adjusting for the independent effects of adulthood BMI, on measures of AUC insulin (β−0.155; 95% CI −0.282, −0.0286; *p*=0.0163) and FG levels (β=−0.0730; 95% CI −0.128, −0.0184; *p*=8.77×10^−3^).

## Discussion

We have performed genetic association analyses and MR to assess the independent causal relationships between BMI recalled from childhood and actual adulthood BMI and the risk of developing type 2 diabetes, as well as their effects on insulin-related traits measured in fasting oral glucose tolerance tests and intravenous glucose tolerance tests. Our measure of childhood BMI provided a more powerful genetic measure than previous work, based upon a combination of self-recall categories and known summary statistics from the 1958NCDS for measured childhood BMI [5].

We found that a higher BMI in childhood, once separated from higher BMI in adulthood, was protective for measures of both insulin secretion and sensitivity, including FG levels and an insulin sensitivity index. We note that associations with lower insulin secretion when not corrected for insulin sensitivity are consistent with a protective effect, because, in people without diabetes, a higher insulin sensitivity results in a reduced need for insulin secretion. One possible explanation is that higher adiposity in childhood stimulates differentiation of cells that are important for insulin sensitivity and secretion, such as adipocytes and beta cells. There is evidence more adipocytes are present in people without diabetes compared to those with diabetes but having the same BMI [21], and that people with higher BMIs but without diabetes have more beta cells [22]. However, the evidence that higher childhood BMI has a protective effect on measures of insulin secretion and sensitivity was not reflected in a conclusive association with protection from type 2 diabetes. This difference may be due to differences in power between measures of continuous traits and binary disease traits, although the type 2 diabetes sample sizes were larger than those for the intermediate traits. Additionally, this effect is relative to other people who may have changed BMI between childhood and adulthood; for example, it may be that the change in BMI is the true risk factor, resulting in a higher BMI in childhood appearing less damaging than a lower BMI in childhood, the latter of which suggests a greater relative increase in adulthood, which is in keeping with observational studies [22, 23]. It is also possible that the genetic variants with stronger associations with childhood BMI result primarily in higher muscle or non-fat mass components of growth. Associations with higher muscle mass may result in higher insulin sensitivity and help explain our findings. However, recent work suggests that the childhood genetic variants used in a previous study [4], which overlap strongly with the genetic variants we used, were good measures of fat mass in children at ages 9–18 years [24]. Crucially, they found that the genetic variants were more strongly associated with adiposity than lean mass in childhood to the beginning of adulthood [24]. That study showed that the variants derived from the UK Biobank childhood BMI self-recall variable are more strongly associated with adiposity than lean mass as measured by dual-energy x-ray absorptiometry at ages 9, 13, 15 and 25 years. Their data showed convincingly that the higher childhood BMI genetic score consistently leads to higher adiposity at several time points in childhood after the adiposity rebound at age 4–5 years, and that these effects are stronger with fat mass than lean mass, although both are present. The associations were also consistent with trajectories of BMI in childhood. More precisely, the childhood BMI genetic instrument was associated with consistently stronger effects on the imaging-based measures than the adulthood genetic instrument at age 9, 13, 15 and 18 years, with a childhood BMI genetic risk score one standard deviation higher than the mean being associated with approximately 8% higher fat mass compared with 1–2% higher lean mass. The adulthood genetic instrument had a stronger effect on fat mass than the childhood instrument by the age of 25 years. It is possible that the likely insulin-sensitising effects of the 1–2% higher lean mass at several times points between the ages of 9 and 18 years offset the insulin resistance effects of the 8% higher fat mass. However, we think this is unlikely because we know that a higher fat mass leads to slightly higher lean mass due to the load-bearing effects of the extra weight, as seen with the *FTO* variant [25]. Importantly, the adulthood BMI genetic instrument is associated with a proportionally similar increase in lean mass for each standard deviation increase in fat mass, and we know that this genetic instrument is associated with lower insulin sensitivity. Whilst we cannot rule it out, we therefore think it unlikely that non-fat mass effects and different trajectories of growth are influencing our results. However, because of these uncertainties, we stress that our work should not lead to any change in clinical practice during childhood, early life or adulthood, and more work is needed to identify the biological mechanisms that could be driving these associations. Nonetheless, adulthood BMI was found to have a consistently risk-increasing/damaging effect on all traits studied, regardless of whether the independent genetic liability to childhood BMI was corrected for, acting as a positive control.

There are a few notable limitations to this study. First, our measure for childhood BMI is derived from a categorical measure that was recalled many decades after the event. To attempt to overcome this limitation, we performed validation in both the 1958NCDS and against external GWAS of BMI measured in childhood, and found that our genetic measures are more strongly predictive of the chronologically relevant phenotypes. We were unable to independently certify that the genetic variants that we used as a proxy of childhood BMI were associated with early-life adiposity, as opposed to (for example) growth and lean mass. We also acknowledge the limitations related to an MR study, in which fully satisfying the three fundamental assumptions (genetic relevance and independence, and no horizontal pleiotropy) is rarely achieved. For example, an MR analysis assumes that the genetic variant does not affect the outcome other than via the exposure: this is unlikely to be consistently the case when considering genetic variants that increase the odds of having type 2 diabetes and BMI if the variant (for example) raised insulin sensitivity independently. There was also some evidence of pleiotropy for three of our analyses using the MR–Egger test.

In summary, our data provide initial evidence that higher fat mass in childhood leads to protective effects, i.e. improvements in insulin sensitivity and reduced need for insulin secretion, in adulthood. A potential explanation is the beneficial effects of exposure to the metabolic challenges of higher adiposity in early, more plastic, stages of life compared with the likely damaging effects of large increases in adiposity between childhood and adulthood.

## Supplementary Information

Below is the link to the electronic supplementary material.

Supplementary file1 (PDF 296 KB)

Supplementary file2 (XLSX 176 KB)

## Data Availability

Data cannot be shared publicly because of data availability and data return policies of the UK Biobank. Data are available from the UK Biobank for researchers who meet the criteria for access to datasets of the UK Biobank (http://www.ukbiobank.ac.uk).

## Abbreviations

1958NCDS: 1958 National Child Development Study
EGG: Early Growth Genetics
GIANT: Genetic Investigation of ANthropometric Traits
METSIM: Metabolic Syndrome in Men
MAGIC: Meta-Analyses of Glucose and Insulin-related traits Consortium
FG: Fasting glucose
FI: Fasting insulin
GWAS: Genome-wide association study
MAF: Minor allele frequency
MR: Mendelian randomisation
MVMR: Multivariable Mendelian randomisation
