# Supplementary material for: Genetic evidence that high BMI in childhood has a protective effect on intermediate diabetes traits, including measures of insulin sensitivity and secretion, after accounting for BMI in adulthood
Source: Diabetologia. 2023 Jun 6;66(8):1472–80. doi: 10.1007/s00125-023-05923-6 (PMC10317883; doi:10.1007/s00125-023-05923-6)
Supplement: Supplementary file 1 — Supplementary file1 (PDF 296 KB) [file 125_2023_5923_MOESM1_ESM.pdf]

# Electronic Supplementary Methods

## Deriving Childhood BMI

We first calculated an estimate of the parameters (shape and scale) describing a gamma distribution of BMI at age 10 based in the participants of the 1958 National Child Development Study. A gamma distribution was chosen due to the known skewness of BMI [1]. Each of the 441,762 individuals within the UK Biobank (UKB) with a self-recalled body size at age 10 were then assigned a value of childhood BMI based on the parameters of this distribution, resulting in an approximation of childhood BMI in a population of size equal to that of the UKB which does not take into account the recall of BMI in childhood (i.e. it is unanchored to the self-recall variable).

The unanchored UKB childhood BMI distribution was then split into three truncated distributions (smaller, normal, larger), of size equal to the three categories describing the self-recall variable. We then estimated the parameters describing each of these three truncated distributions as individual gamma distributions - we chose not to model truncated distributions because of the assumed overlap of the edges of the categories.

Each participant's self-recall childhood BMI was then used to assign them to one of the three sub-distributions, from which a childhood BMI is randomly assigned from the modelled parameters of the sub-distribution. The culmination of these values then describes a simulation of what is henceforth referred to as childhood BMI in the UKBB, which is anchored to the self-recall variable described above.

# Supplementary Results

## Estimating A Continuous Measure of Childhood BMI in the UK Biobank

The parameters describing the gamma distribution that best fit the 1958NCDS BMI at age 11 variable were k = 53.5 (shape parameter) and $\theta$ = 0.269 (scale parameter). There were 145,378, 226,176, and 70,207 UKB individuals who self-recalled as being 'thinner than their peers, the same size

as their peers, or 'plumper' than their peers at age 10 respectively. Figure 1 then demonstrates how the distribution sampled from the 1958NCDS parameters above is split according to the sizes of the three groups, and in Figure 2 we re-sample these sub-populations as unique distributions. The cumulation of the three distributions resulted in the anchored childhood BMI estimation shown in Figure 3. The final distribution shown in Figure 3 had k=53.7 and $\theta$ = 0.267, which is approximately identical the initial input parameters used.

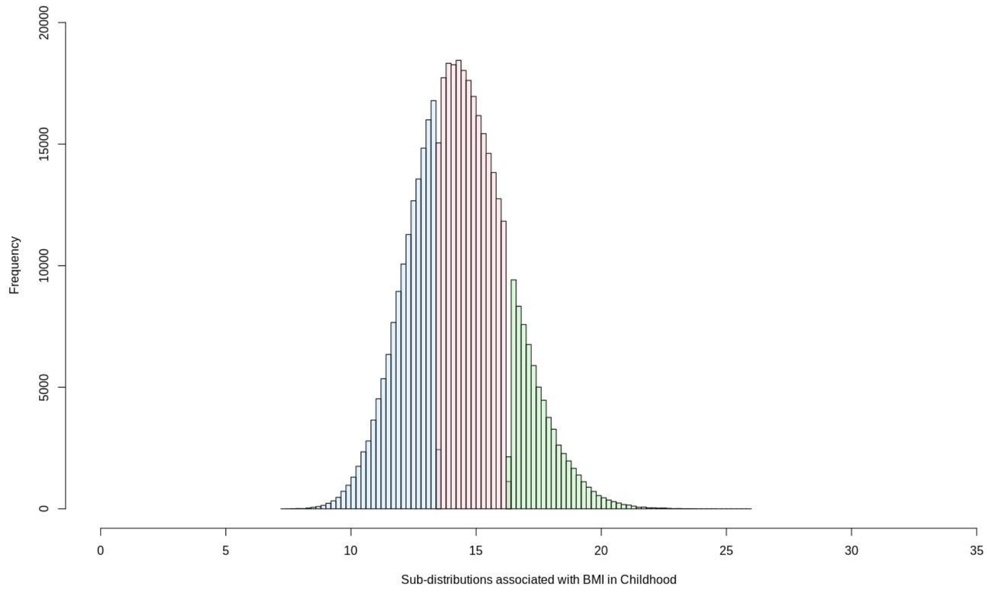


**Fig 1.** Sub-populations of UK Biobank participants trichotomised from overarching simulation of childhood BMI from 1958NCDS parameters. Individuals who recalled themselves to be thinner, the same as, or plumper than their peers are coloured in blue, red and green respectively.

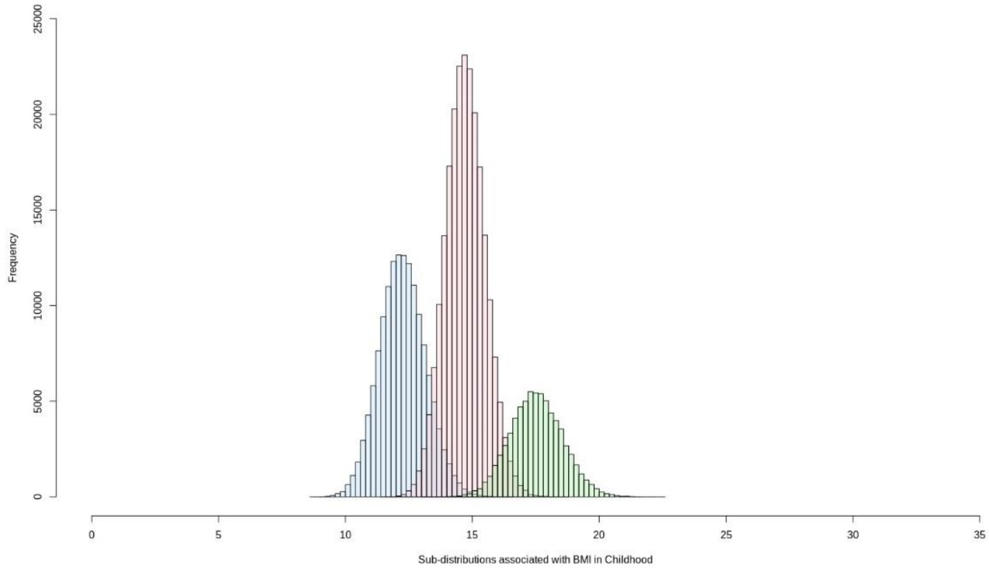


**Fig 2.** Sub-populations re-sampled from trichotomised childhood BMI distributions. Individuals who described themselves as thinner, the same as, or plumper than their peers are coloured in blue, red and green respectively.

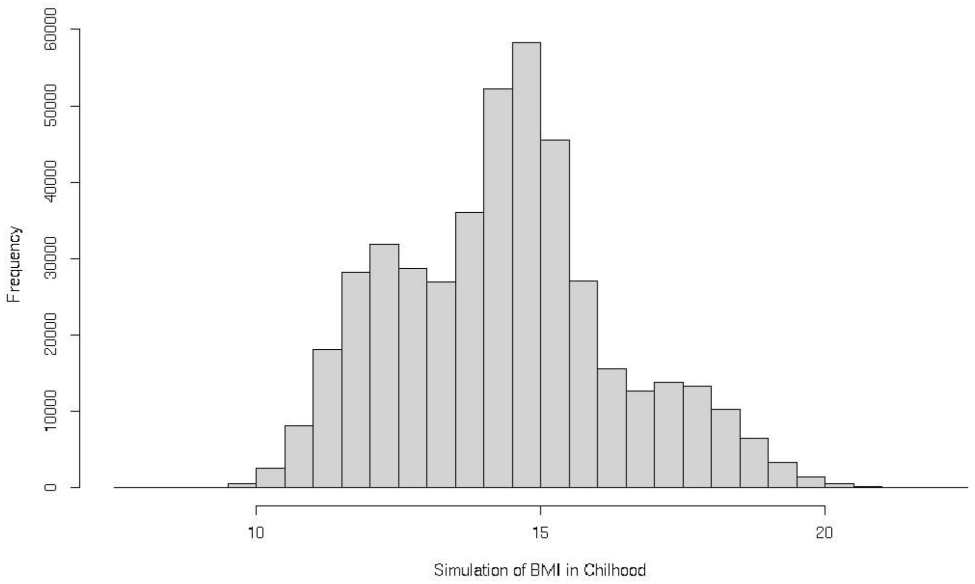


**Fig 3.** Emulated BMI in Childhood from 1958NCDS summary statistics and categorical self-recall data of body size

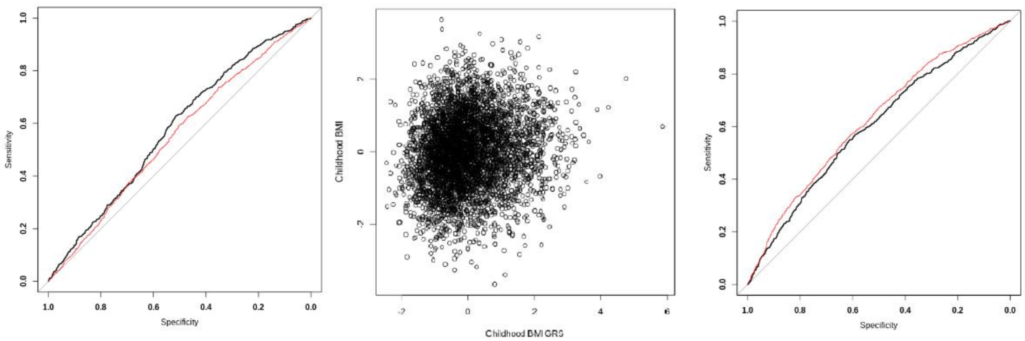


**Fig 4.** ROCs for childhood BMI (left) and adulthood BMI (right) polygenic scores, assessed against a binary outcome of being > 1s.d from the mean of age 11 BMI (black) or age 44 BMI (red) in the 1958 National Child Development Study, and a scatter plot of childhood BMI GRS (x-axis) against rank-inverse normalised childhood BMI at age 11 (middle)

# Supp References


1. Muth´en B, Asparouhov T. Growth mixture modeling with nonnormal distributions. Statistics in Medicine. 2015;34(6):1041–1058. doi:10.1002/sim.6388.
